# Clinical and Molecular Impact of Advanced Platelet-Rich Fibrin on Pain, Swelling, and Distal Periodontal Status of Mandibular Second Molars After Mandibular Third-Molar Extraction

**DOI:** 10.3390/medicina60122062

**Published:** 2024-12-14

**Authors:** Ada Stefanescu, Irina-Georgeta Sufaru, Iulia Chiscop, Fabian Cezar Lupu, Cristian Martu, Bogdan Oprisan, Kamel Earar

**Affiliations:** 1 Faculty of Dental Medicine, “Dunarea de Jos” University, Al. I. Cuza Street 35, 800216 Galati, Romania; 2 Faculty of Dental Medicine, Grigore T. Popa University of Medicine and Pharmacy, Universitatii Street 16, 700115 Iasi, Romania; 3 Mechanical Engineering, Mechatronics and Robotics Department, “Gheorghe Asachi” Technical University of Iasi, 700050 Iasi, Romania; 4 Faculty of Medicine, Grigore T. Popa University of Medicine and Pharmacy, Universitatii Street 16, 700115 Iasi, Romania

**Keywords:** A-PRF, clinical parameters, cytokines, oral surgery, pain, tumefaction

## Abstract

*Background and Objectives*: This study aimed to evaluate the role of A-PRF (advanced platelet-rich fibrin) in the enhancement of wound healing and protecting the periodontal health of mandibular second molars after the extraction of mandibular third molars. Additionally, the study assessed the levels of pro-inflammatory cytokines in the gingival crevicular fluid (GCF) of mandibular second molars as markers of inflammation. *Materials and Methods*: Twenty-five systemically healthy adult patients with bilateral removal of impacted mandibular third molars were included. Each patient received A-PRF in one extraction site, while the contralateral site served as a control. Periodontal parameters of the adjacent second molar, including probing depth (PD) and clinical attachment level (CAL), were measured in distal–vestibular (DV) and distal–lingual (DL) sites. Pain, swelling, and overall healing were subjectively evaluated. Levels of tumor necrosis factor-alpha (TNF-α), interleukin 1 beta (IL-1β), and interleukin 6 (IL-6) in the GCF were analyzed. Evaluations occurred at baseline and three months post-surgery. *Results*: A-PRF significantly improved PD (from 4.69 ± 0.61 mm to 3.85 ± 0.34 mm in DV, and from 4.71 ± 0.65 mm to 3.79 ± 0.27 mm in DL, respectively) and CAL (from 2.41 ± 0.25 mm to 1.82 ± 0.21 mm in DV, and from 2.40 ± 0.36 mm to 1.75 ± 0.19 mm in DL, respectively) of the adjacent second molar, compared to control sites, three months post-surgery. Pain and swelling scores were notably lower on the 7th postoperative day in the A-PRF group. A-PRF also reduced pro-inflammatory cytokines in GCF, significantly more than in control sites, at three months post-surgery. *Conclusions*: A-PRF enhances the periodontal and inflammatory status of adjacent teeth and wound healing after the extraction of mandibular third molars.

## 1. Introduction

Third-molar extraction is a common surgical intervention in dental practice, and the postoperative inflammatory complications associated with it are well-known in the specialized literature [1]. Pain, edema, and trismus are among the most commonly reported symptoms after this procedure, and significantly affect patients’ quality of life [2]. Indeed, these adverse effects can negatively influence recovery and patient comfort in the postoperative period [3].

Localized periodontal damage to the mandibular second molar is a common complication following the extraction of impacted mandibular third molars, particularly in cases of partial eruption. Several mechanisms contribute to this damage. The elevation of mucoperiosteal flaps and manipulation of soft and hard tissues during third-molar extraction can traumatize the periodontal tissues of the adjacent second molar, leading to increased probing depth (PD), clinical attachment loss (CAL), and localized inflammation [4]. Partially erupted third molars often harbor bacterial biofilm and promote plaque accumulation due to their difficult-to-clean position [5]. This can lead to chronic inflammation and periodontal pockets on the distal aspect of the second molar, predisposing it to periodontal disease and tissue destruction even prior to extraction. Studies have shown that partially erupted third molars are frequently associated with increased probing depths and loss of clinical attachment on the adjacent tooth [6].

However, therapies have been developed and proposed to manage these effects. Marx et al. [7] presented platelet-rich plasma (PRP) as a groundbreaking method utilizing blood-derived platelet preparations. They highlighted its superior angiogenic and osteogenic capabilities compared to the standard clot that forms in the alveoli following tooth extraction. To extract PRP, they centrifuged 400 to 450 mL of a patient’s whole blood at 5600 rpm, using citrate phosphate dextrose as an anticoagulant.

After extensive research, Jo et al. [8] modified the PRP technique. The original method used citrate phosphate dextrose as an anticoagulant, with calcium gluconate and bovine thrombin serving as coagulants. The process was initiated by centrifuging 9 mL of the patient’s blood at 900 rpm for 5 min, producing three separate fractions: red blood cells, buffy coat, and white blood cells. The top two fractions were subsequently collected and subjected to further centrifugation at 1500 rpm for 15 min, leading to the extraction of the upper layer of PPP, which left only 2 mL of PRP in the tube.

On the other hand, Anitua et al. [9] introduced a new concept of autologous blood derivative called PRGF (Plasma Rich in Growth Factors), which accelerates the healing process. A fibrin clot was generated from the patient’s blood by a single 8-min centrifugation at 1850 rpm using 5 mL plastic tubes containing sodium citrate anticoagulant. In addition to the challenges associated with the use of coagulants and anticoagulants, there are also risks associated with the use of bovine thrombin, which can cause coagulopathies [10].

Advanced platelet-rich fibrin (A-PRF) represents an evolution of PRF, prepared using a lower centrifugation speed and modified protocols, which results in an enhanced fibrin matrix with higher concentrations of leukocytes and platelets [11]. This composition allows for a prolonged and more effective release of growth factors, such as vascular endothelial growth factor (VEGF), transforming growth factor-beta (TGF-β), and platelet-derived growth factor (PDGF) [12], which are critical for wound healing and tissue regeneration.

Even though it is recognized that A-PRF contains a higher concentration of growth factors and autologous cells than traditional PRF, no study has been performed to date to evaluate the effect of this therapy on healing wounds, particularly its potential influence on inflammatory markers. In fact, to date, research on the actual clinical significance of A-PRF is limited, and a small number of studies have been conducted in this regard [13,14,15].

Despite these promising findings, there is limited clinical evidence on the effects of A-PRF in specific dental procedures, such as the extraction of mandibular third molars. Mandibular third-molar extractions are often associated with damage to the periodontal health of adjacent teeth [16]. This damage can manifest as increased probing depths, loss of clinical attachment, and heightened inflammatory responses, ultimately contributing to periodontal instability.

Thus, the main aim of this study was to investigate the therapeutic potential of A-PRF in enhancing the wound healing process, measured by periodontal parameters of mandibular second molars, as well as pain and tumefaction. In addition, inflammatory markers in the gingival crevicular fluid (GCF) were analyzed to understand the biological mechanisms underlying its therapeutic effects. The null hypothesis of the study was that the application of A-PRF in post-extraction sockets of impacted mandibular third molars would not result in significant differences in wound healing, periodontal parameters (probing depth and clinical attachment loss), pain, swelling, or proinflammatory cytokine levels, compared to control sites without A-PRF application.

## 2. Materials and Methods

### 2.1. Study Population

This interventional, prospective, randomized, split-mouth study was performed on 25 systemically healthy adult patients, female and male, who were indicated for extraction of mandibular third molars and had intact mandibular second molars. Patients with bilateral removal of impacted mandibular third molars were included. Only vertical impactions classified as Class II, Position B, according to Pell and Gregory’s classification [17], were selected.

The exclusion criteria from the study were represented by the following:

- The presence of systemic diseases;
- Patients who smoked;
- The presence of acute local or systemic infections;
- Use of medications that may affect periodontal or systemic health (e.g., immunosuppressants and anticoagulants);
- Pregnant patients or nursing mothers;
- Patients with drug allergies;
- Lack of two mandibular molars;
- Mandibular second molar with coronal destruction;
- Mandibular second molar with direct or indirect restoration.

All participants underwent standard pre-operative blood tests, including a complete blood count (CBC). Patients with abnormal results or systemic conditions that could affect healing were excluded from the study.

The study was conducted in accordance with the Declaration of Helsinki and approved by the University Ethics Committee of “Dunarea de Jos” University Galati (protocol code 242/CEU/28.05.2024).

### 2.2. Therapeutic Procedures

All patients underwent bilateral extraction of the impacted mandibular third molar. The molar was surgically removed in a single session.

Two sites of the mandibular second molar were selected as experimental sites for the measurement of periodontal parameters:

1. The distal–vestibular site (DV);
2. The distal–lingual site (DL).

These two sites were selected owing to their proximity to the extracted tooth.

Each patient underwent A-PRF placement in one post-extraction site, while the other post-extraction site served as a control. The therapeutic site (A-PRF application) and control site were randomized using a sealed-envelope method. Prior to surgery, 25 opaque, sealed envelopes were prepared, each containing a randomly generated assignment (left or right) for the A-PRF application site. These assignments were generated using a computer-based randomization tool to ensure allocation concealment.

An independent researcher not involved in the clinical assessments or surgical procedures opened the envelopes sequentially during the procedure. This process ensured that the surgeon and the patient were blinded to the randomization until the A-PRF was applied. The control site was the contralateral extraction socket, which received no additional therapeutic intervention.

All surgeries were performed according to a standardized technique and involved strict application of asepsis measures to minimize the risk of infections before and during the surgical procedure.

An inferior alveolar nerve block was performed for local anesthesia, along with a buccal infiltration using a 4% articaine with epinephrine 1:200,000 solution (Ubistesin Normal 3M Espe, 3M Deutschland GmbH, Neuss, Germany).

The procedure included creating a distal extension envelope flap, for which a full-thickness mucoperiosteal flap was elevated to facilitate tooth extraction. After the extraction, the socket was thoroughly rinsed with a sterile 0.9% saline solution. The control group sites were then primarily closed with 4.0-gauge polyglycolic acid (PGA) simple sutures (D-Tek 4.0, Demophorius, Limassol, Cyprus), without any additional material.

A specialized operator took two tubes of venous blood, each 10 mL in capacity, from a sterile source without additives or anticoagulants to obtain A-PRF. The tubes were centrifuged for 8 min at a rotational speed of 1500 rpm (Premiere XC-2000, C&A Scientific, Sterling, VA, USA). The finished product, A-PRF, was then transferred to a PRF Box^f^ and compressed into a plug approximately 10 mm in diameter and 5–8 mm in thickness and inserted into the extraction socket to ensure complete site coverage. After placing A-PRF in the socket, 4.0-gauge PGA simple sutures (D-Tek 4.0, Demophorius, Limassol, Cyprus) were used to close the extraction sites.

After surgery, all patients underwent a postoperative management protocol, which included the administration of an oral antibiotic (Augmentin Duo every 12 h, seven days) (GlaxoSmithKline, London, UK) and a nonsteroidal anti-inflammatory drug (ibuprofen 400 mg every 8 h, five days) (Reckitt Benckiser, Slough, UK).

In addition to drug treatment, patients were given detailed instructions on postoperative oral hygiene and prescribed a 0.2% chlorhexidine solution (Curasept ADS 220, Curaden AG, Kriens, Switzerland) for mouth-rinsing twice daily for 14 days. All patients were instructed to strictly follow postoperative recommendations to ensure a quick and efficient recovery and to minimize the risk of complications.

Patients were monitored for any complications, including infection, dry socket, excessive bleeding, or sensory disturbances, during the postoperative period. No such complications were observed in either the A-PRF or the control group.

### 2.3. Clinical and Paraclinical Evaluations

#### 2.3.1. Clinical Parameters

The measures used to evaluate the results included probing depth (PD), clinical periodontal attachment loss (CAL), and subjective assessment of pain, swelling, and healing degree. PD and CAL were measured in the distal areas of mandibular second molars at the DV (buccal) and DL (lingual) sites. PD and CAL were assessed utilizing a UNC 15 periodontal probe (Henry Schein, Gillingham, Kent, UK). PD was ascertained by measuring the gingival margin from the base of the periodontal pocket. Concurrently, CAL was documented as the distance from the enamel–cementum junction to the base of the periodontal pocket.

A clinical assessment of these parameters was carried out by a single examiner (I.-G.S.) without awareness of the treatment method. Prior to the commencement of the study, the examiner underwent a calibration process. Intra-examiner calibration was performed on a subset comprising 10% of the patients, with duplicate measurements of PD and CAL taken 48 h apart. The examiner was deemed calibrated when a statistically significant correlation and a statistically insignificant difference were noted between the duplicate measurements (r = 0.85 for PD and 0.90 for CAL).

The evaluations were carried out at two distinct time points, at the beginning of the study (T0), i.e., before the tooth extraction, and three months after the intervention (T1), to monitor the evolution and effects of the treatment.

The Wound Healing Index (WHI) [18] was recorded by the same investigator who evaluated the periodontal status of the selected teeth 7 and 14 days after surgery to evaluate the patients’ postoperative evolution. Within this index, each case received a score between 1 and 3, depending on the degree of healing observed (Table 1).

Pain and swelling were recorded using visual scores at 7 and 14 days (Table 2).

To assess the level of discomfort felt by the patients, they were asked to complete a visual analog scale (VAS) [19], which allowed them to express the intensity of the pain they were experiencing. This standardized method of pain assessment allowed patients to indicate their degree of discomfort on a line of predetermined length, from one extreme representing no pain to the other indicating the most intense pain felt. Clinical observations formed the basis for assessing facial swelling and healing progress.

#### 2.3.2. Evaluation of Inflammatory Markers in Gingival Crevicular Fluid (GCF)

Tumor necrosis factor alpha (TNF-α), interleukin 1 beta (IL-1β), and interleukin 6 (IL-6), which are known for their significant roles in inflammation and the body’s immune response, were quantified in gingival crevicular fluid (GCF).

This fluid was collected from the deepest periodontal pocket identified in the adjoined teeth. After selecting the appropriate site, the area was carefully isolated using sterile cotton rolls. Once the area was prepared, it was gently air-dried. Then, a sterile paper cone was gently inserted into the identified periodontal pocket, allowing it to interact with the fluid for 30 s. After sampling, the paper cone was carefully extracted and placed in a sterile Eppendorf tube.

We used the sandwich ELISA method to analyze the pro-inflammatory cytokines according to the specific protocol provided by the kit manufacturer. This test involves using monoclonal and polyclonal antibodies, which bind to different epitopes of the antigen, ensuring accurate and specific detection. Cones impregnated with gingival crevicular fluid were placed in 450 μL of phosphate-buffered saline and agitated for 30 min to release cytokines. We then divided the solution into aliquots and stored the samples at −80 °C to ensure their stability and integrity until the analysis.

Cytokine analysis was performed by constructing a standard curve using the standards provided in the assay kit. Protein concentrations in our samples were then calculated against this standard curve.

We dispensed 100 μL of our diluted standards and samples in duplicate into wells coated with antibodies specific to each cytokine. The plate was incubated at room temperature for 1 h, after which the wells were washed three times to remove excess solution. The next step involved adding 100 μL of conjugate solution containing a specific protein antibody and peroxidase conjugate. The plate was then incubated at room temperature for 2 h. After rewashing the wells, we added 100 μL of substrate solution and incubated the plate for 20 min to allow color development. The reaction was stopped by adding 50 μL of stop solution, and the absorbance was measured at 450 nm using a plate reader.

TNF-α, IL-1β, and IL-6 concentrations were determined and normalized to pg/mL gingival crevicular fluid. Molecular assessment was performed at baseline and repeated at 3 months to monitor the evolution of inflammation and immune response.

### 2.4. Sample Size Calculation

**Figure 1:**
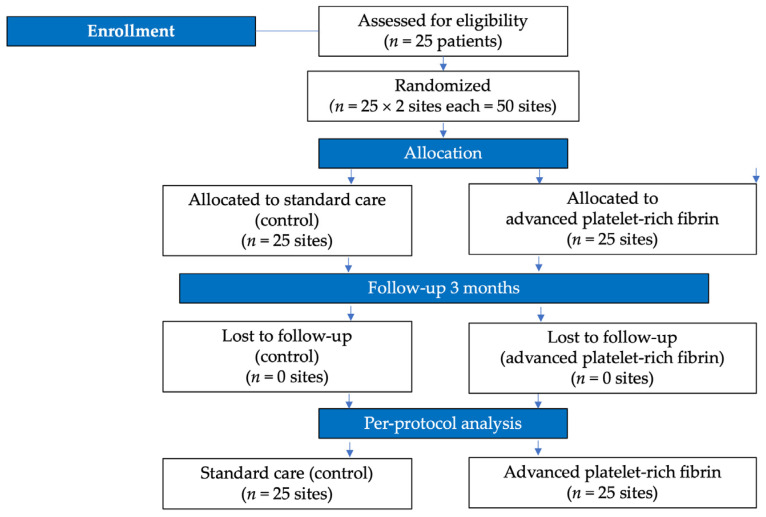
Study’s flowchart.

The sample size calculation was performed to ensure sufficient statistical power for detecting significant differences in the primary outcome, the CAL. We anticipated a mean difference of 1 mm in CAL improvement between treatment site groups, with an estimated standard deviation of 0.5 mm. Using an alpha significance level of 0.05 and a power of 0.90, it was determined that a minimum of 21 sites per treatment group would be required to detect significant differences [20]. To account for potential subject loss throughout the study or dropouts during follow-up, we included an additional 20% in the sample size calculation, leading to a determination of 25 subjects. The study’s flowchart is depicted in Figure 1.

### 2.5. Statistical Analysis

Continuous data such as probing depth (PD) results, clinical attachment loss (CAL), and inflammatory molecule concentrations are presented as mean (x –) ± standard deviation (SD), reflecting the mean and dispersion of the data.

Conversely, categorical data, such as visual scores for swelling and healing, are presented as numbers and percentages to illustrate the distributions of these categories within the sample.

To represent the pain score on the visual analog scale (VAS), we calculated the median and interquartile range (IQR), which indicate both the central value and the data’s variability. We also computed the confidence interval for the VAS, offering insights into the precision of the measurements.

A mixed ANOVA test was utilized to evaluate the relationship between the intervention (compared to control) and the outcome of interest across various time points.

The significance of fixed effects (*p*-values) was evaluated using the Kenwood–Roger (KR) approximation.

Pain, swelling, and healing scores were analyzed using the Wilcoxon paired signed-rank test, suitable for non-normally distributed data.

Paired *t*-tests with Tukey’s HSD correction were conducted as a post hoc test to pinpoint significant differences among groups. All hypothesis tests were conducted at a 0.05 significance level, employing a two-tailed approach to explore potential differences in both directions.

## 3. Results

### 3.1. Demographic Results

We screened 25 patients eligible for inclusion in the study; all of the initially included subjects returned for periodic re-evaluations.

The study population’s gender distribution was represented by 12 male patients (48.00%) and 13 female patients (52.00%), with a mean age of 31 ± 2.73 years. Of these, nineteen subjects came from the urban environment (76.00%) and six from the rural environment (24.00%).

### 3.2. Clinical Results

There was no statistical difference between the values of the clinical parameters in the sites of the two groups at baseline.

At the 3-month assessment, the control sites group demonstrated a slight increase in mean probing depth values for both sites (DL and DV), although this did not reach the threshold of clinical significance (*p* = 0.137) (Table 3).

A statistically significant decrease in PD was observed postoperatively for the A-PRF sites group at 3 months, compared to the preoperative baseline (Table 3), for both sites (from 4.69 ± 0.61 mm to 3.85 ± 0.34 mm for DV sites; from 4.71 ± 0.65 mm to 3.79 ± 0.27 mm for DL sites). Values also differed significantly between site groups at T1 (*p* < 0.001 for both sites, DL and DV) (Table 3).

Clinical periodontal attachment loss followed the same trend as PD. CAL showed a slight increase in mean values in both sites (DL and DV) for the control group without reaching the threshold of clinical significance (*p* = 0.137) (Table 3).

The CAL demonstrated a statistically significant postoperative decrease for the A-PRF group at three months, compared to the preoperative baseline, for both sites (from 2.41 ± 0.25 mm to 1.82 ± 0.21 mm for DV sites; from 2.40 ± 0.36 mm to 1.75 ± 0.19 mm for DL sites) (Table 3).

In addition, values differed significantly between groups postoperatively at 3 months (*p* < 0.001 for both sites, DL and DV) (Table 3). The visual score comparison indicated that on the 7th postoperative day, pain (*p* = 0.01) and swelling (*p* = 0.03) were significantly reduced in the A-PRF group compared to the control group (Table 4).

**Figure 2:**
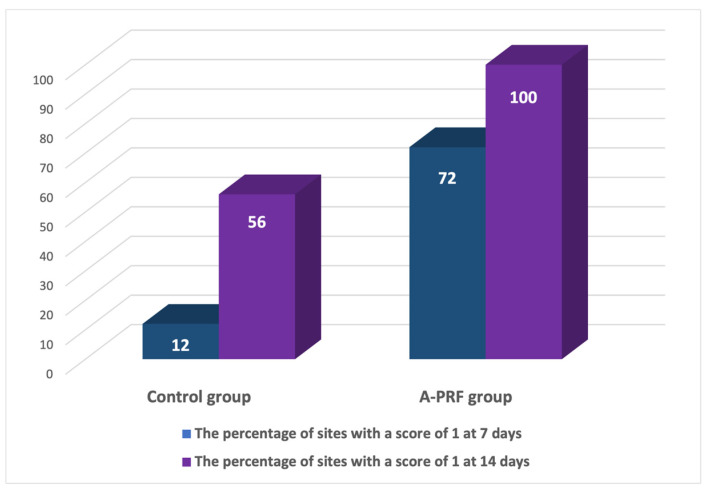
Percentage distribution of healing index 1 postoperatively at 7 and 14 days.

Figure 2 shows the wound healing index (WHI) postoperatively at 7 and 14 days. At seven days, results showed significant healing in the A-PRF group, where 56.00% of sites showed perfect healing, with a WHI score of 1. In comparison, only 12.00% of sites in the control group achieved this performance (*p* < 0.001).

At 14 days, observations showed that all sites (100%) treated with A-PRF showed perfect healing, and the WHI score was 1, while in the control group, the percentage was 72.00%. This difference was statistically significant (*p* < 0.001), highlighting the effectiveness of A-PRF treatment in promoting rapid and complete healing of postoperative wounds.

It is worth mentioning that no complications were reported postoperatively at 7 or 14 days, indicating a favorable safety profile of the intervention used. These findings suggest that A-PRF can significantly improve wound healing after surgery, providing promising results without immediate adverse events.

### 3.3. Molecular Results

Baseline analysis of TNF-α revealed similar values for both intervention groups (*p* = 0.642) (Table 5).

The third analyzed marker, IL-6, initially showed similar values between the groups. Three months post-intervention, IL-6 values decreased significantly only for the A-PRF group (from 104.22 ± 2.21 pg/mL to 73.401 ± 5.25 pg/mL, *p* < 0.05) (Table 5).

For the control and A-PRF groups, the Δ values were 6.81 ± 2.20 pg/mL and 32.26 ± 4.37 pg/mL, respectively (*p* < 0.001).

Following the quantification of TNF-α three months post-intervention in the control group, the mean value showed a slight decrease, without reaching the threshold of statistical significance: from 14.01 ± 2.24 pg/mL to 13.85 ± 0.75 pg/mL, with a Δ = 0.96 ± 0.12.

In the A-PRF group, we observed a significant decrease in this inflammatory marker (from 13.20 ± 2.25 pg/mL to 10.80 ± 1.51, with Δ value = 3.19 ± 0.31). The decreases were also more significant for the A-PRF group (*p* < 0.05) (Table 5).

IL-1β levels were similar at baseline for both groups. At three months post-intervention, however, IL-1β values were significantly lower for patients in the A-PRF group compared to patients in the control group, with Δ values being 23.82 ± 5.32 pg/mL and 97.43 ± 10.29 pg/mL for the control and A-PRF groups, respectively (*p* < 0.001) (Table 5).

## 4. Discussion

The principal objective of this study was to evaluate the effects of A-PRF on distal periodontal damage of the adjacent second molar after the extraction of mandibular third molars in conjunction with the evaluation of postoperative healing. It marks the first randomized controlled trial assessing A-PRF’s clinical and molecular effectiveness as a biomaterial for periodontal healing of mandibular second molars after third-molar extractions. The study is not limited to evaluating the clinical effects of the treatment but also aims to examine the impact on pro-inflammatory molecular markers. The biochemical analysis of pro-inflammatory cytokines in GCF offered molecular insights not typically addressed in studies focusing on A-PRF. This dual approach provides deeper insights into the mechanisms by which A-PRF can generate beneficial effects on periodontal damage, reduce inflammation, and promote healing in a high-risk area.

The extraction of molar teeth is one of the most frequent surgical procedures in dental practice globally [21]. Like any medical procedure, molar extraction comes with the associated risk of complications, either intra-operatively or postoperatively. The most common complications include post-extraction alveolitis, postoperative infectious processes, and inferior alveolar nerve lesions [21]. Understanding and properly managing the risks associated with molar extraction is essential to ensure an optimal surgical outcome and prevent postoperative complications that can affect patients’ quality of life. Nevertheless, no complications, such as infection, dry socket, excessive bleeding, or sensory disturbances, were observed in either study group during the postoperative period.

The periodontal parameters of the adjacent second molar, including probing depth (PD) and clinical attachment loss (CAL), are critical indicators of periodontal health following mandibular third-molar extractions [16]. Surgical trauma during third-molar extractions can lead to inflammation and periodontal damage of the adjacent teeth, manifesting as increased PD and CAL, and elevated pro-inflammatory cytokines [22].

In this study, A-PRF demonstrated significant efficacy in minimizing these adverse effects. The distal reduction in PD and CAL observed in the A-PRF group after three months suggests that A-PRF enhances soft-tissue healing and protects the periodontal integrity of adjacent teeth. These notable reductions could not be seen in the control group, which did not receive the same therapy. These results are clinically significant because preserving the periodontal health of the second molar is a critical consideration in third-molar extractions, particularly when surgical trauma can compromise long-term periodontal stability [23]. Moreover, these findings are particularly significant, as the current literature provides limited insight into the effects of A-PRF on periodontal outcomes for adjacent teeth following extraction.

Suwondo et al. [24] compared periodontal tissue regeneration using A-PRF and PRF in treating infrabony pockets. Twenty infrabony pockets were divided into two groups: OFD + A-PRF (10) and OFD + PRF (10). PD and RAL were measured on days 0, 30, and 90, while bone height was assessed using CBCT on days 0 and 90. The OFD + A-PRF group showed significantly greater reductions in PD and RAL than the OFD + PRF group, with no difference in bone height between groups.

The findings suggest that A-PRF enhances periodontal regeneration more effectively than PRF in infrabony pocket treatment. A-PRF variant is rich in growth factors, including platelet-derived growth factor AA (PDGF-AA), epidermal growth factor (EGF), and insulin-like growth factor 1 (IGF-1), which promote wound healing and stimulate cell growth. Pitzurra et al. [11] demonstrated the advantages of A-PRF+ over classic L-PRF in promoting fibroblast healing in preclinical research.

Upadhyay et al. [25] compared PRF and A-PRF in the treatment of periodontal infrabony defects (IBDs), using clinical and radiographic evaluations. Twenty-eight patients were divided into Group A (PRF) and Group B (A-PRF). The comparison revealed no statistically significant differences between the groups. The authors concluded that both materials individually produced promising results, with the PRF group (Group A) showing better outcomes in bone fill and the A-PRF group (Group B) excelling in soft-tissue healing.

Another study compared the effects of advanced platelet-rich fibrin (A-PRF+) and enamel matrix derivative (EMD) with open flap debridement (OFD) alone in grade II molar furcation sites, focusing on clinical outcomes and wound healing parameters [26]. Seventeen patients were randomly assigned to A-PRF+, EMD, or OFD groups, with both patients and examiners blinded. A minimally invasive microsurgical approach was used for all treatments. Clinical measurements were taken at baseline and 6 months post-op, and healing was assessed using the wound healing index at days 3, 7, 14, and 42. The case series (RCT design) indicates a slight advantage of A-PRF+ over EMD and OFD in regressing furcation grade II to grade I; however, A-PRF+ demonstrated delayed early wound healing compared to EMD and OFD. Nonetheless, the clinical situation was different from our study.

Zahid and Nadershah [27] conducted an evaluation of A-PRF effects on bone regeneration and postoperative outcomes following the extraction of impacted third molars. This study involved the measurement of PD, gingival recession (GR), and CAL at baseline, one month, and three months post-procedure. Additionally, pain, swelling, and healing assessments were performed on day seven in a split-mouth design involving ten patients. Three months post-extraction, the results showed no significant differences between the test group, in which A-PRF was used, and the control group, which did not receive this additional treatment [27]. As a result, the authors concluded that using A-PRF provided no additional benefit in reducing probing depth, reconstructing connective tissue attachment, and minimizing gingival recessions compared to natural healing.

We observed that on the 7th day after surgery, the pain and swelling scores in the A-PRF-treated group were significantly lower than those in the control group. These findings are in agreement with the results of other studies, such as those by Kumar et al. in 2015 [28] and He et al. in 2017 [29]. Using A-PRF may reduce the pain symptoms and swelling associated with surgical tooth-extraction procedures. The results also showed that at 14 days post-operation, all sites treated with A-PRF had a healing score of 1, indicating complete healing. This aspect emphasizes the effectiveness of A-PRF in accelerating the healing process and promoting a quick and complication-free recovery.

Pereira et al. [30] evaluated the effects of A-PRF+ on the healing of upper third-molar post-extraction sockets. The study was conducted using a split-mouth design, and with 16 subjects. The alveoli on the test side were filled with A-PRF+, while the control side was maintained with blood clot. The clinical analysis revealed gradual reductions in pain, edema, and bleeding and improved soft-tissue repair in both groups at 90 days compared to the 7-day post-surgical follow-up, without any significant differences between groups. Moreover, the use of A-PRF+ did not show a clinical benefit in the healing of post-extraction sockets for upper third-molars. Nevertheless, the study focused on upper third-molars, which differ anatomically and in healing dynamics from mandibular third molars.

In the study by Zahid and Nadershah [27], post-extraction alveolar augmentation with an A-PRF clot significantly improved postoperative pain and tumefaction within seven days postoperatively. A study by Gupta and Agarwal [31] found that using A-PRF reduced post-extraction discomfort, compared to a control group that did not receive this augmentation treatment. The groups had no significant differences regarding post-extraction swelling or trismus on the first postoperative day. Still, on the third day, patients in the A-PRF group reported substantial improvements in pain and the ability to open their mouths wide.

These findings suggest that A-PRF may effectively manage postoperative symptoms and accelerate the recovery process after tooth extraction. By reducing discomfort and improving temporomandibular joint function, A-PRF may significantly benefit patients experiencing the adverse effects of dental surgery.

In our study, the use of A-PRF led to a significant decrease in the levels of pro-inflammatory cytokines TNF-α, IL-1β, and IL-6 postoperatively at 3 months, and this reduction was more pronounced than in the control group. It is important to note that elevated levels of these cytokines are often present in patients undergoing active inflammation or dealing with systemic conditions [32]. This can extend the inflammatory phase of post-extraction wound healing, as the heightened local production of pro-inflammatory cytokines may impair the tissue-regeneration process.

Pro-inflammatory cytokines, such as IL-1β, IL-6, IL-17, and TN-Fα, trigger a feed-forward mechanism, compelling neighboring cells to produce additional pro-inflammatory cytokines and chemokines. These neighboring cells may include those of mesenchymal origin, such as fibroblasts in soft connective tissue [33]. Chronic inflammation leads to tissue destruction and disrupts the cellular processes essential for tissue regeneration [34]. Kargarpour et al. [35] observed that PRF lysates significantly suppressed the inflammatory response to TNFα and IL1β.

The significant decrease in these cytokines observed in the A-PRF-treated group suggests that using this biomaterial may modulate the inflammatory response and accelerate healing. A-PRF preparations are rich in growth factors that can stimulate periosteal cell proliferation, indicating that they can serve as a reservoir for delivering specific growth factors to the application site [36]. Therefore, A-PRF may have substantial potential in managing postoperative inflammation and promoting faster and more effective post-extraction wound healing [37]. Furthermore, the association between reduced cytokine levels and improved periodontal parameters highlights the role of inflammation modulation in promoting periodontal healing. Future studies could expand on these findings by exploring long-term periodontal outcomes and the potential of A-PRF in other high-risk scenarios, such as patients with pre-existing periodontal disease.

One significant research direction involves comparing the concentrations of inflammatory cytokines in a patient’s peripheral blood with their concentrations in blood concentrates, such as A-PRF, following centrifugation. This comparison could provide a better understanding of how blood concentration influences the cytokine profile of patients and could provide valuable clues about the potential of A-PRF to modulate these inflammatory responses.

Without a doubt, further research is necessary to clarify the relationship between pro-inflammatory cytokine levels and post-extraction alveolar healing and evaluate the potential risks of blood-concentrate treatments in patients with systemic conditions marked by chronic inflammation.

This study had several limitations, including its small sample size, which limited the ability to generalize the results. Studies on a larger sample are needed to obtain more robust conclusions and better validate the effects of A-PRF on bone regeneration.

While this study specifically focused on periodontal parameters of mandibular second molars as teeth adjacent to the extractional site, the findings also suggest broader implications for A-PRF in reducing post-extraction complications, such as dry socket or alveolar bone loss. This determination was informed by the study’s objective to evaluate the effect of A-PRF on periodontal healing rather than alveolar bone regeneration. Future research should build on these results by exploring long-term periodontal outcomes and the roles of A-PRF in other clinical scenarios. It is also important to acknowledge the potential interplay between socket healing and the improvement of periodontal parameters. Future studies should integrate socket-healing evaluations to further clarify their relationship with periodontal health outcomes

Moreover, extending the follow-up period to 6 months would enhance the statistical power of a study, allowing it to identify differences in outcomes related to A-PRF’s impacts on bone healing and regeneration. This longer follow-up interval would allow more detailed observation of patients’ evolution and provide more conclusive information about A-PRF therapy’s long-term efficacy.

Continuous development and optimization are essential because tissue engineering methods, including A-PRF, PRP, PRGF, and others, are relatively novel in medicine and dentistry. This development requires extensive and rigorous clinical trials in large groups of patients so that robust data can be collected and more detailed information can be obtained about these technologies’ efficacy, safety, and potential.

By addressing these issues, the medical and scientific community can advance the complex understanding of how blood concentrates and other tissue-regeneration techniques can influence post-extraction healing and be integrated into clinical practice to improve treatment outcomes and the patient experience.

## 5. Conclusions

Compared to blood clots, A-PRF significantly reduced pain and swelling in the postoperative period and promoted better tissue regeneration, including a more favorable periodontal attachment. These benefits were also supported by a more significant decrease in the inflammatory cytokines, suggesting a positive influence of A-PRF on the healing process. However, long-term randomized control trials with larger study groups are needed to validate these findings and adequately obtain more robust results.

## Figures and Tables

**Table 1:** The interpretations of Wound Healing Index (WHI) scores.

Score	Signification
1	Excellent healing, with no signs of gingival edema, erythema, suppuration, patient discomfort, or flap dehiscence. This category indicates an optimal recovery with no notable complications.
2	Good healing but with minor signs of gingival edema, erythema, patient discomfort, or flap dehiscence. However, the absence of suppuration indicates a positive course of healing, with possible minor complications.
3	Deficient healing, characterized by signs such as significant gingival edema, erythema, patient discomfort, flap dehiscence, or suppuration. This category suggests a suboptimal healing which may require additional interventions to prevent further complications and promote full wound recovery.

**Table 2:** Assessments of swelling and pain.

Parameter	Score	Variables
Pain	0	Absent
	1	Shallow
	2	Moderate
	3	Severe
Tumefaction	0	Absent
	0.1–3.0 cm	Shallow
	3.1–6.0 cm	Moderate
	6.1–10 cm	Severe

**Table 3:** Comparison of periodontal parameter values across sites and treatment groups at the two time-points of assessment.

Site	Time	PD		CAL	
		Control Sites (*n* = 25)	A-PRF Sites (*n* = 25)	Control Sites (*n* = 25)	A-PRF Sites (*n* = 25)
DV	T0	4.73 ± 0.82	4.69 ± 0.61	2.45 ± 0.32	2.41 ± 0.25
	T1	4.95 ± 1.14	3.85 ± 0.34 ^ab^	2.82 ± 0.59	1.82 ± 0.21 ^ab^
DL	T0	4.78 ± 0.79	4.71 ± 0.65	2.48 ± 0.39	2.40 ± 0.36
	T1	4.89 ± 1.22	3.79 ± 0.27 ^ab^	2.97 ± 0.88	1.75 ± 0.19 ^ab^

Values are expressed as mean (mm) ± standard deviation; DV: distal–vestibular; DL: distal–lingual; PD: probing depth; CAL: clinical periodontal attachment loss; ^a^: *p* < 0.05 in intra-group comparisons T1 versus T2; ^b^: *p* < 0.05 in comparisons between groups at T1 and T2, respectively.

**Table 4:** Swelling and pain scores in study groups.

Parameter	Score	Control Sites (*n* = 25)	A-PRF Sites (*n* = 25)	*p* Value
Tumefaction	0	3	13	0.03
	1	15	10	
	2	5	2	
	3	2	0	
Pain (VAS)		5 [4.25–6.75]	2.5 [2–4.75]	0.01

Tumefaction is presented as number of subjects (*n*); pain is presented as median/IQR.

**Table 5:** Cytokine levels in the two study groups at baseline and postoperatively at 3 months.

Parameter	Control Sites (*n* = 25)			A-PRF Sites (*n* = 25)		
	T0	T1	Δ	T0	T1	Δ
TNF-α	14.01 ± 2.24	13.85 ± 0.75	0.96 ± 0.12	13.20 ± 2.25	10.80 ± 1.51 ^ab^	3.19 ± 0.31 ^c^
IL-1β	331.15 ± 16.29	309.02 ± 12.52	23.82 ± 5.32	316.07 ± 14.26	224.31 ± 11.42 ^ab^	97.43 ± 10.29 ^c^
IL-6	111.88 ± 3.25	107.36 ± 3.15	6.81 ± 2.20	104.22 ± 2.21	73.401± 5.25 ^ab^	32.26 ± 4.37 ^c^

Values are expressed as mean ± standard deviation (pg/ml); ^a^ *p* < 0.05 in the same group, T1 vs. T0; ^b^ *p* < 0.05 between groups at the same time point; ^c^ *p* < 0.05 (Student or Mann Whitney *T*-test for non-uniformly distributed values).

## Data Availability

The data used to support the findings of this study are available from the corresponding author upon reasonable request.
